# Effects of Acute Piano Performance With Blood Flow Restriction on Upper Limb Muscle and Perceptual Response in Pianists

**DOI:** 10.7759/cureus.63074

**Published:** 2024-06-24

**Authors:** Tomohiro Yasuda, Yumi Sato, Toshiaki Nakajima

**Affiliations:** 1 Exercise Physiology, Seirei Christopher University, Hamamatsu, JPN; 2 Child Education, Okazaki Women’s University, Aichi, JPN; 3 Cardiovascular Medicine, Dokkyo Medical University, Mibu, JPN

**Keywords:** piano, muscle fatigue, muscle thickness, kaatsu, blood flow restriction training

## Abstract

Background: Long-term and prolonged piano performance does not provide essential skeletal muscle training benefits while increasing the risk of injury to the upper extremities. Unlike high-intensity exercise training, moderate blood flow restriction (BFR) training has been found to improve neuromuscular mechanisms with a variety of physical exercises (machine, elastic band, walking, electrical stimulation, and body weight).

Aim and methods: We investigated the physiological and perceptual responses related to piano performance with or without BFR based on acute responses of neuromuscular mechanisms. Student or professional pianists (n=7) performed the "Revolutionary Etude" on the piano with (Piano-BFR) and without (Piano-Ctrl) BFR. During the Piano-BFR performance, 150-180 mmHg of cuff pressure was applied around the most proximal region of both arms as a moderate BFR.

Results: Changes in upper limb girth, muscle thickness, and hand grip strength were measured before and immediately after the performance. After the performance, perceptual and other responses were recorded. Immediately after the performance, the Piano-BFR condition induced greater changes in girth (forearm and upper arm), muscle thickness (forearm), and handgrip strength than the Piano-Ctrl condition. Piano-BFR was (p<0.01) higher than Piano-Ctrl on eight questions regarding perceptual response (upper arm fatigue and difficulty playing the piano). Piano performance with BFR was revealed to increase upper extremity muscle size and fatigue in pianists after playing.

Conclusion: Piano performance with BFR was revealed to increase upper extremity muscle size and fatigue in pianists after playing. The effect of BFR on neuromuscular mechanisms on piano performance was greater in the forearm than in the upper arm.

## Introduction

It has generally been presumed that the more time one spends practicing piano performance, the more one can improve one's upper extremity piano playing skills, but there are concerns about three major diseases (tendonitis, carpal tunnel syndrome, and focal dystonia) due to excessive practice [1-4]. Although it is well known that traditional high-intensity exercise training (>70% 1-repetition maximum: 1RM) is effective in improving neuromuscular function in skeletal muscles, physical activity during piano playing is primarily low intensity (at the 20-30% level) even in the most active upper limb muscle groups [5-7]. Given these observations, long-term and prolonged piano performance does not provide essential skeletal muscle training benefits while increasing the risk of injury to the upper extremities.

High-intensity exercise training is a useful technique for adapting the neuromuscular function, but there have been concerns about the large stresses on the joints as well as cardiovascular systems and the restricted movements involved in training [8]. On the other hand, since approximately 20 years ago, moderate blood flow-restricted (not complete occlusion of blood flow) training (up to 30% 1RM), known as KAATSU training, has been known to cause muscle hypertrophy and has no adverse effects on joints or the cardiovascular system [9,10]. BFR training uses a special KAATSU cuff worn at the base of the limbs to properly restrict blood flow with pressure [11]. This training is a noninvasive technique, although it does cause blood flow restriction (BFR). Its safety has been confirmed by previous questionnaires, blood tests, and venous compliance testing in young, elderly, or patients [8,12,13]. In addition, unlike high-intensity exercise training that uses weights or free-weight machines, BFR training improves neuromuscular mechanisms not only by using weights or free-weight machines but also by using a variety of physical exercises (machines, rubber bands, walking, electrical stimulation, and body weight) [14-16].

Therefore, we hypothesized that piano performance in combination with BFR may be useful for improving neuromuscular mechanisms in the upper limb. In this study, we investigated the physiological and perceptual responses to piano performance with or without BFR based on acute responses to neuromuscular function.

## Materials and methods

Participants

This research was approved by the Ethics Committee of Seirei Christopher University. We prospectively recruited human participants for the study. Seven students or professional pianists volunteered to participate in the study (aged 16-56 (two males and five females)). They passed the inclusion criteria of at least 10 years of piano experience and the ability to play Chopin's "Revolution" and the exclusion criteria mentioned in the previous BFR study (subjects with resting blood pressure >160/100 mmHg, anemia, cerebrovascular disease, myocardial infarction, or history of arthroscopic surgery are excluded) so there were seven subjects in the study [10]. One minor (16 years old) was included in this study. Therefore, six adults signed their own consent to participate, and for one minor participant, the child's guarantor signed the consent form. The recruitment period for this study began on November 12, 2021, and the experiment ended on February 17, 2023.

Blood pressure

Before the start of the piano performance, a manchette (Terumo Electronic Blood Pressure Monitor H55, Terumo Corporation, Tokyo, Japan) was wrapped around the participant's left upper arm, and resting blood pressure was measured in the sitting position using the oscillometric method.

Piano performance

**Figure 1:**
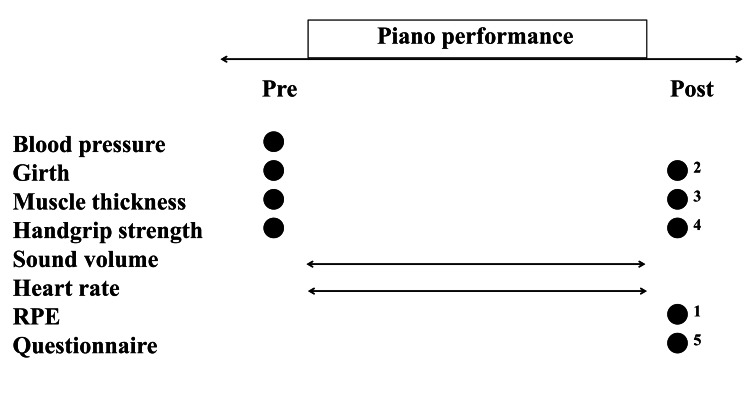
Experimental timeline points at which measurements were taken. ^●^points of each measurement. ^⇔^continuous measurement during the piano performance. After, each measurement was performed as quickly as possible in the order of one to five. BFR, blood flow-restricted; RPE, ratings perceived exertion

A grand piano (YAMAHA C3, Yamaha Music Japan Co., Ltd., Hamamatsu, Japan) in the university music room (length: approximately 11 m and width: approximately 6.5 m) was used (temperature: 23.0±2.9°C, humidity: 45.9±21.2%). The Chopin “Revolutionary Etude” (Op. 10 No. 12, performance time: approximately three minutes) was selected for the piano performance, with (Piano-BFR: maintaining BFR from the beginning to the end of the piano performance) and without BFR (Piano-Ctrl) [17]. Each participant performed Piano-BFR (one trial) or Piano-Ctrl (one trial) in random order at intervals of at least one hour. A summary of the protocol is shown in Figure 1.

Blood flow restriction

At least one week prior to the experiment, participants were accustomed to playing the piano under BFR conditions with a specially designed elastic pneumatic pressure cuff (30 mm wide, KAATSU Master, KAATSU Japan, Tokyo, Japan) at the most proximal position of both arms. The pressure was set at 150-180 mmHg to obtain a moderate BFR. This is because previous studies have reported that this is the optimal BFR level to alter blood flow and energy supply as well as increase muscle activation without interfering with the total work of the upper arm [18-21].

In the Piano-BFR protocol, participants were seated on a chair, and cuffs with a pressure (pressure applied by the cuff to the arm before inflation) of 30 mmHg were tightened on the arms at the most proximal position. The cuffs were then inflated to 150-180 mmHg and rapidly removed after the piano performance to complete each measurement. The time under moderate BFR was approximately four to five minutes, including about three minutes of performance music.

Girth

Forearm length was measured between the ulnar eminence and the radial head, the point 30% proximal to the elbow on each arm was marked with a marker pen, and the girth of both forearms was measured with a tape measure. Similarly, upper arm length was measured between the lateral epicondyle of the humerus and the acromioclavicular process of the scapula, the point 60% distal to the acromioclavicular process was marked with a marker pen, and the girth at the marked point was measured for both arms. Measurements were performed before and immediately after the piano performance, with the participant standing in a relaxed position with elbows extended. 

Muscle thickness

Using B-mode ultrasound (FAMUBO, Seikosha, Tokyo, Japan), muscle thickness (MTH) was measured at three locations on each arm: one forearm (at 30% proximal between the styloid process and the head of the radius) and two upper arms (anterior and posterior 50% region). The measurement was carried out with the participant standing in a relaxed position with elbows extended. A 7.5/10.0 MHz scanning head (4.5 cm length probe) was set on the skin perpendicular to the tissue interface. The scanning head was coated with a water-soluble permeable gel to provide acoustic contact without depressing the dermal surface. The interface between subcutaneous adipose tissue and muscle and the interface from muscle to bone were identified using ultrasound images. The vertical distance from the adipose tissue-muscle interface to the muscle-bone interface was defined as the MTH. Ink markers for the forearm flexor and elbow flexor/extensor muscle groups were used to ensure similar positioning for repeat measurements of MTH. The average of twice for “pre” or “post” was represented as a single data point for statistical analysis, respectively. 

Handgrip strength

Handgrip strength of the left and right before and after piano performance was measured with a factory-calibrated hand dynamometer (TKK 5401, Takei Co., Ltd., Tokyo, Japan). Participants were instructed to hold the dynamometer in an upright position with their arms at their sides and their elbows extended 180° without pressing their arms against their bodies. The handle of the dynamometer was set to a size at which the participants could comfortably hold and exert effort. Participants were instructed to exert maximal effort as quickly as possible and maintain it for one to two seconds. Two attempts were performed, and the better attempt was used for analysis. 

Sound volume

During the experiment, a sound level meter was used to continuously record the volume (Sound Level Metre MK09, Metrek, USA) during each piano performance from ground level and a distance of approximately 1.5 m and 9 m from the grand piano, respectively. The sound level meter readings (noise level) ranged from approximately 40 to 50 dB before each piano performance, and the maximum volume during the piano performance was used as the representative value.

Heart rate

Heart rate during each piano performance was continuously recorded by a fitness watch (A360, POLAR JAPAN Corporation, Tokyo, Japan) worn on the participant's left wrist. The maximum heart rate value during each piano performance was used for analysis.

Rating of perceived exertion

Immediately following each piano performance, a rating of perceived exertion was recorded, which measures the participant's subjective sense of exertion and fatigue on the Borg scale (6-20) [22].

Questionnaire

Participants were asked to answer the following questions immediately after completing the piano performance (except for Q9 and Q10, which were only for the Piano-BFR condition). For each of the above questions, each participant was asked to answer one of the following five options (5: strongly agree, 4: agree, 3: neither agree nor disagree, 2: disagree, and 1: strongly disagree). (Q1) “Has your fingertip strength decreased?” (Q2) “Has your handgrip strength decreased? (Q3) “Did you have difficulty opening your fingertips? (Q4) “Did you have difficulty playing the piano?” (Q5) “Was it difficult to play the piano fast?” (Q6) “Did you have difficulty moving your fingers horizontally?” (Q7) “Did you feel the volume was low when playing the piano?” (Q8) “Do you think the tone during the piano performance was rare?” (Q9) “Do you feel that your fingertips are sensitive after removing the cuff?” (Q10) “Do you feel the increased freedom of movement of your fingers after removing the cuffs?” 

Statistical analysis

The results are presented as the means±standard deviations (SD). Statistical analysis was performed using a two-way analysis of variance (ANOVA) with repeated measures (trials (Piano-BFR vs. Piano-Ctrl)×time (pre vs. post)). Post-hoc testing was performed using a t-test with Bonferroni’s correction when appropriate. Percent changes from before were also compared between the groups using Tukey’s test. All calculations were made with JMP statistical software package v.12 (SAS Institute Inc., Tokyo, Japan). Statistical significance was set at p<0.05. Pre/post-effect sizes (ESs, Cohen’s d) in circumference and handgrip strength were calculated using the following formula: ((post-mean-pre-mean)/pre-SD; d=0.2, small effect; d=0.5, moderate effect; and d=0.8, large effect) [23].

## Results

The coefficient of variation (CV) of girth measurement from test to retest was 0.4% for the forearm and 0.6% for the upper arm. The CV of MTH measurement from test to retest was 3.2% for the forearm and 2.9% and 4.2% for the anterior and posterior upper arms, respectively. The CV of handgrip measurement from test to retest was 2.2%. The sound level meter readings (noise level) ranged from approximately 40 to 50 dB before each piano performance. No signs of pain or discomfort were observed during acclimation to piano performance under the pressure conditions. The piano performance and physical characteristics of the participants are shown in Tables 1, 2. All participants were right-handed. Heart rate and sound volume during piano performance had similar maximum values for both Piano-Ctrl (131±6 BPM and 93.7±1.6 dB) and Piano-BFR (131±6 BPM and 93.5±1.7 dB).

**Table 1:** Characteristics of each participant.

Participant	Age (years)	Occupation		Piano history (years)
#1	16	High school student (music course)		11
#2	20	University student (no music course)		15
#3	56	Professional pianist		52
#4	41	Professional pianist/university teacher		35
#5	45	Professional pianist/university teacher		42
#6	49	Professional pianist/university teacher		42
#7	46	Professional pianist/university teacher		42

**Table 2:** The physiological characteristics and clinical data in pianists (n=7). Data are given as mean (SD). aUpper arm, anterior upper arm; BMI, body mass index; BP, blood pressure; MTH, muscle thickness; L, light; pUpper arm, posterior upper arm; R, right; SD, standard deviation

Variable		Mean (SD)	Range
Physiological characteristics			
Age, years		38.9 (15.0)	16-56
Standing height, cm		165.3 (12.0)	154-183
Body weight, kg		65.9 (22.3)	49.0-110.0
BMI, kg/m^2^		23.6 (4.8)	19.5-32.8
Resting systolic BP, mmHg		122 (16)	101-140
Resting diastolic BP, mmHg		73 (18)	51-101
Resting heart rate, BPM		78 (13)	58-98
Length			
Finger length (L), cm		21.5 (1.5)	19.1-23.3
Finger length (R), cm		21.5 (1.4)	19.6-23.2
Forearm length (L), cm		24.1 (2.5)	22.0-29.0
Forearm length (R), cm		24.1 (2.5)	22.0-29.0
Upper arm length (L), cm		30.8 (2.8)	28.0-35.5
Upper arm length (R), cm		30.8 (2.8)	28.0-35.5
Girth			
Forearm girth (L), cm		24.4 (4.1)	20.7-32.4
Forearm girth (R), cm		24.8 (3.7)	21.2-32.0
Upper arm girth (L), cm		28.3 (5.7)	23.2-40.0
Upper arm girth (R), cm		28.7 (6.0)	23.3-40.5
Muscle thickness (MTH)			
Forearm MTH (L), cm		1.80 (0.35)	1.44-2.22
Forearm MTH (R), cm		1.88 (0.24)	1.67-2.20
aUpper arm MTH (L), cm		2.33 (0.40)	2.01-3.03
aUpper arm MTH (R), cm		2.47 (0.59)	2.00-3.50
pUpper arm MTH (L), cm		1.95 (0.29)	1.66-2.43
pUpper arm MTH (R), cm		2.05 (0.35)	1.55-2.40
Strength			
Handgrip (L), kg		30.6 (6.8)	21.4-39.2
Handgrip (R), kg		30.7 (7.4)	22.9-41.4

After the piano performance, Piano-BFR showed significant increases (p<0.05) in forearm girth (left 2.4%, right 3.4%) and upper arm girth (left 1.6%, right 2.7%) compared to baseline, while Piano-Ctrl showed only in left forearm girth significantly increased (0.9%) compared to baseline (Table 3). Effect sizes of the differences in girth between pre- and post-piano performance were all larger for Piano-BFR (0.08-0.22) than for Piano-Ctrl (0.00-0.05). After the piano performance, the muscle thickness of the left and right forearm increased significantly (13.2%, p<0.01 and 7.4%, p<0.05) in the Piano-BFR group, while no significant changes were observed in any muscle thickness in the Piano-Ctrl group (Table 3). The effect sizes of the differences in girth between pre- and post-piano performance were all larger for Piano-BFR (0.08-0.22) than for Piano-Ctrl (0.00-0.05). The effect sizes of the differences in muscle thickness (MTH) of the forearm between pre- and post-piano performance were larger for Piano-BFR (0.59-0.76) than for Piano-Ctrl (0.06-0.14). After the piano performance, handgrip strength decreased significantly (-7.5%, p<0.05) only in the left arm of Piano-BFR. The effect sizes of the differences in handgrip strength between pre- and post-piano performance were both larger for the Piano-BFR group (left: 0.11, right: 0.29) than for the Piano-Ctrl group (left: 0.05, right: 0.09). When the rate of force development (RFD, at 0-200 msec) of handgrip strength was measured on three participants in this study, Piano-BFR (left: pre (18.4 kg) vs. post (9.3 kg); right: pre (19.8 kg) vs. post (12.3 kg)) showed a more pronounced decrease in RFD than Piano-Ctrl (left: pre (18.2kg) vs. post (16.8kg); right: pre (19.1 kg) vs. post (19.1 kg)) [24].

**Table 3:** Changes in forearm, upper arm girth, and MVC of the piano performance at pre- and immediately post-piano performance. Values are presented as means and SDs. **Different from pre-piano performance, p<0.01. *Different from pre-piano performance, p<0.05. MVC, maximum voluntary contraction; aUpper arm, anterior upper arm; pUpper arm, posterior upper arm; SD, standard deviation

		Piano-BFR (n=7)				Piano-Ctrl (n=7)			
		Pre	Post	% change	Effect size	Pre	Post	% change	Effect size
Girth, cm									
Forearm	Left	24.5 (4.0)	25.1 (4.0)^**^	2.4	0.16	24.5 (4.1)	24.7 (4.1)^**^	0.9	0.05
	Right	24.8 (3.7)	25.6 (3.8)^**^	3.4	0.22	24.8 (3.8)	24.9 (3.9)	0.2	0.02
Upper arm	Left	28.2 (5.8)	28.7 (5.8)^**^	1.6	0.08	28.2 (5.6)	28.4 (5.9)	0.4	0.03
	Right	28.6 (6.0)	29.3 (5.5)^**^	2.7	0.11	28.6 (5.8)	28.6 (5.7)	0.1	0.00
Muscle thickness, cm									
Forearm	Left	17.3 (3.7)	19.5 (3.7)^**^	13.2	0.59	18.0 (3.5)	17.5 (2.8)	-2.1	0.14
	Right	18.6 (1.9)	20.1 (3.4)*	7.4	0.76	18.8 (2.4)	18.7 (2.1)	-0.5	0.06
aUpper arm	Left	22.7 (5.0)	24.3 (4.0)	8.1	0.31	23.3 (4.0)	24.3 (4.3)	4.0	0.23
	Right	24.4 (7.0)	26.0 (5.0)	8.7	0.22	24.7 (5.9)	24.6 (6.6)	-0.4	0.01
pUpper arm	Left	19.8 (4.0)	21.1 (3.3)	8.0	0.35	19.5 (2.9)	20.2 (3.3)	3.7	0.26
	Right	20.8 (4.1)	20.9 (2.9)	1.4	0.01	20.5 (3.5)	21.5 (2.0)	5.6	0.27
Handgrip strength, kg									
MVC	Left	29.9 (8.4)	28.9 (7.8)	-2.4	0.11	29.9 (7.3)	30.3 (8.4)	0.5	0.05
	Right	31.4 (8.8)	28.8 (7.3)*	-7.5	0.29	30.8 (7.7)	31.5 (8.6)	2.1	0.09

Piano-BFR was more than two points higher than the score in the Piano-Ctrl (p<0.01) on eight questions regarding some perceptual responses (upper arm fatigue and difficulty playing the piano) (Table 4). All participants (n=7) reported that their fingertip nerves were sharpened immediately after the Piano-BFR performance was completed and the cuff released. 

**Table 4:** Perceptual responses after the piano performance with (Piano-BFR) or without BFR (Piano-Ctrl). Values are presented as means and SDs. For each of the above questions, each participant was requested to answer one of the following five options (5: strongly agree, 4: agree, 3: neither agree nor disagree, 2: disagree, and 1: strongly disagree). ^##^Different from Ctrl, p< 0.01. BFR, blood flow restriction; Q, questionnaire; SD, standard deviation

		Piano-BFR	Piano-Ctrl
Q1	Decreased in fingertip strength	4.0 (0.0)^##^	1.4 (0.8)
Q2	Decreased in handgrip strength	3.4 (0.8)^##^	1.4 (0.8)
Q3	Hard to open fingertips	3.7 (0.8)^##^	1.7 (0.8)
Q4	Difficulty in piano performance	4.1 (0.4)^##^	2.0 (1.2)
Q5	Difficulty in playing fast	4.0 (0.6)^##^	1.7 (0.8)
Q6	Difficulty of moving the finger horizontally	3.9 (0.7)^##^	1.4 (0.8)
Q7	Low volume in piano performance	4.0 (0.8)^##^	1.7 (0.8)
Q8	Scarcity of tones	4.0 (0.8)^##^	1.6 (0.8)
Q9	Sensitive fingertip sensation after removing the cuffs	4.6 (0.5)	-
Q10	Increased freedom of fingers movement after removing the cuffs	4.3 (0.8)	-

## Discussion

We hypothesized that the method of piano performance with BFR would have a significant effect on the neuromuscular function of the upper limb. In this study, we examined how piano performance under BFR conditions affects skeletal muscle and perceptual responses. The results of the effects on neuromuscular function were found to be similar to the acute responses in a previous study [21].

A previous study on BFR showed that immediately after low-intensity knee extension exercise, the leg girth (an indicator of muscle swelling) was more pronounced under BFR conditions than under non-BFR conditions [25]. In addition, a single low-intensity BFR bench press exercise acutely increased muscle size in both the BFR triceps brachii muscle and the non-BFR pectoralis major muscle; after 12 training sessions, the muscle cross-sectional area of the triceps brachii and pectoralis major muscles increased significantly in the BFR bench press group [20]. Therefore, the swelling of muscle cells due to BFR training may contribute significantly to the anabolic effect of BFR [26]. In the present study, upper arm girth increased by 1.7% to 2.7% immediately after the piano performance in the Piano-BFR group. Previous studies on BFR resistance exercise have shown 3.5% (75 reps/four sets and 6.4% (75 reps/four sets) increases in upper arm girth immediately after arm curl exercise (unpublished data) [18,19]. 

Resistance exercises in the previous study were single-joint exercises, activating one or two agonist muscles, while this study used exercises that activated the various working muscles required for piano performance. In fact, the measurement of MTH at various sites in the upper limb showed a significant increase after piano performance only in the left forearm under BFR conditions, indicating a large regional difference in muscle activity. 

Thus, the contribution of each muscle during physical activity differs greatly between piano performance and resistance exercise. Furthermore, the forearm girth showed a large increase compared to the upper arm girth, confirming that the forearm is particularly important for the movements required during piano performance. Based on these considerations, it can be speculated that piano performance with BFR has a particularly large effect on improving neuromuscular function (e.g., muscle hypertrophy and increased muscle strength) in the forearm rather than the upper arm.

In the present study, the duration of the piano performance was approximately three minutes, a time setting similar to that of resistance exercise (four sets of total exercise time) used in many previous studies [10,19]. Previous studies have reported that longer rest intervals between physical performances with BFR attenuate muscle fatigue, and the BFR condition in this study was an environment in which fatigue was easily induced [19,21]. However, as mentioned above, since piano playing is a physical exercise that activates various upper limb muscles (especially the movement of the muscle group that controls fingertip movement, which is complex and intermittent), changes in circumference (muscle swelling) and muscle fatigue during piano playing are likely to be distributed to the muscles of each finger. Therefore, the changes can be expected to be small compared to resistance exercises in previous studies that focused on local (simple and continuous) areas. On the other hand, the heart rate measured during the piano performance in this study was the same as that during the walk training with restricted venous blood flow from the leg muscle, suggesting that Piano-BFR may promote not only local muscle size increase but also stimulation of cardiorespiratory function as a whole-body exercise [14].

In this study, the maximum heart rate was at the same level regardless of BFR. In the case of skilled piano players, piano performance is carried out while moving the entire body, so even Piano-Ctrl is likely to have reached the level of aerobic exercise. On the other hand, BFR was limited to the muscle groups mainly in the fingers and forearms that were directly related to keyboard striking in piano playing, and the effect of stimulation of these local muscle groups may have remained an extremely low phenomenon compared to the whole-body exercise. In a previous study, heart rate was reported to increase when BFR was combined with BFR for the lower limb muscle groups active as the primary muscles in walking [14]. The difference between the results of the previous study and the present study would be whether the muscle activity in the blood flow-restricted area is due to a larger or smaller muscle group. Therefore, it is likely that the combined use of BFR in piano performance did not lead to a further increase in the heart rate.

In addition, the results of RFD indicate that Piano-BFR can induce fatigue in the neuromuscular mechanism due to piano playing in a shorter time than Piano-Ctrl and that practice with Piano-BFR may prevent the three major diseases (tendonitis, carpal tunnel syndrome, and focal dystonia) associated with excessive piano practice [2-4]. 

In this study, differences between right and left limbs were observed in girth change, handgrip strength, and rating of perceived exertion. The "Chopin's Revolution" adopted in this study is characterized by the requirement of continuous smooth movements of the left hand and intermittent movements of the right hand that use multiple fingers simultaneously [27]. Since performing a musical instrument often requires asymmetrical body movements, it can be speculated that the left and right limbs are affected differently according to the muscular activity of each arm. Such differences between the left and right limbs are unlikely to occur with resistance exercise and may be the cause of the observed differences in the effects of resistance exercise and music performance.

This study focused on the effects on muscle morphology and function. From the perspective of effect size, Piano-BFR did not reach the point of exerting a significant influence on muscle morphology or muscle function. On the other hand, a previous study reported the possibility that resistance exercise with BFR could modulate cortical motor excitability due to changes in sensory feedback via group III and group IV afferents [28]. They also indicated that this is an acute sign of neuromuscular adaptation that underlies muscle strength changes after resistance exercise with BFR. Therefore, the acute signs of neuromuscular adaptation supporting post-exercise muscle strength changes may also be observed in Piano-BFR. Further research is needed on Piano-BFR and neuromuscular adaptation.

The present study has several limitations. First, the sample size was small due to the difficulty of recruiting pianists with the ability to play Chopin's Revolution. Future studies using robust experimental designs with large sample sizes should be conducted to validate the results of this study. Second, the present study had a short-term effect, and the long-term effects are only speculative. Third, since all the participants in this study were right-handed, the influence of the dominant arm could not be eliminated regarding the left-right difference in piano performance with BFR. Therefore, further research is needed to validate these findings.

This article was posted on 12 April 2023, at preprint sever Research Square. 

## Conclusions

The hypothesis that the method of Piano-BFR would have a significant effect on the neuromuscular function of the upper extremity was substantiated by the increase in muscle thickness and circumference of the upper extremity and the increase in fatigue (muscle size and perceptual responses).

Piano playing with BFR has a particularly significant effect on improving neuromuscular mechanisms (e.g., muscle hypertrophy and increased muscle strength) in the forearm rather than the upper arm.

In this study, differences between left and right limbs were observed in the assessment of girth change, grip strength, and perceptual response. These differences between left and right limbs are unlikely to occur with resistance exercise and may be due to differences in the effects of resistance exercise and music performance. Differences between left and right limbs are less likely to occur with resistance exercise and may be the cause of the observed differences in the effects of resistance exercise and music performance.
